# ATP7B knockout disturbs copper and lipid metabolism in Caco-2 cells

**DOI:** 10.1371/journal.pone.0230025

**Published:** 2020-03-10

**Authors:** Sarah Guttmann, Oksana Nadzemova, Inga Grünewald, Malte Lenders, Eva Brand, Andree Zibert, Hartmut H. Schmidt

**Affiliations:** 1 Medizinische Klinik B für Gastroenterologie und Hepatologie, Universitätsklinikum Münster, Münster, Germany; 2 Gerhard-Domagk-Institute of Pathology, University Hospital Muenster, Münster, Germany; 3 Department of Nephrology, Hypertension and Rheumatology, Internal Medicine D, University Hospital Muenster, Münster, Germany; University of Saskatchewan, CANADA

## Abstract

Intestinal cells control delivery of lipids to the body by adsorption, storage and secretion. Copper (Cu) is an important trace element and has been shown to modulate lipid metabolism. Mutation of the liver Cu exporter *ATP7B* is the cause of Wilson disease and is associated with Cu accumulation in different tissues. To determine the relationship of Cu and lipid homeostasis in intestinal cells, a CRISPR/Cas9 knockout of *ATP7B* (KO) was introduced in Caco-2 cells. KO cells showed increased sensitivity to Cu, elevated intracellular Cu storage, and induction of genes regulating oxidative stress. Chylomicron structural protein *ApoB48* was significantly downregulated in KO cells by Cu. Apolipoproteins *ApoA1*, *ApoC3* and *ApoE* were constitutively induced by loss of *ATP7B*. Formation of small sized lipid droplets (LDs) was enhanced by Cu, whereas large sized LDs were reduced. Cu reduced triglyceride (TG) storage and secretion. Exposure of KO cells to oleic acid (OA) resulted in enhanced TG storage. The findings suggest that Cu represses intestinal TG lipogenesis, while loss of *ATP7B* results in OA-induced TG storage.

## Introduction

The absorption of lipids and essential trace elements, including copper (Cu), is predominantly mediated by specific cells of the small intestine. Dietary intake and processing of lipids has to be considered in metabolic diseases of Cu homeostasis, like Wilson disease (WD) and Menke disease (MD) [1, 2]. Excess Cu is toxic and usually manifests with increased liver Cu load and Cu excretion. Low Cu is frequently associated with impairment of various biochemical processes and growth inhibition. The molecular mechanism that governs uptake and intracellular metabolism of Cu and lipids by intestinal cells is not fully understood. Infant rhesus monkeys revealed decreased Cu retention suggesting a reduced intestinal Cu absorption following Cu exposure [3]. MD patients suffer from Cu deficiency, caused by mutation of Cu transporter *ATP7A*, a ubiquitously expressed Cu exporter, resulting in deficiency of Cu in the body due to Cu accumulation in the enterocyte. In contrast, WD patients are characterized by Cu overload of various tissues, prominently liver and brain, due to mutation of Cu transporter *ATP7B* [4]. High accumulation of Cu in the liver is followed by increased oxidative stress (e.g. *HMOX1*, *MT1*, and *SOD1*), mitochondrial damage and reduced ceruloplasmin (*CP*) release [5, 6]. In liver, Cu is mainly imported by Cu transporter 1 (*CTR1*) and divalent metal transporter 1 (*DMT1*), although a minor role of multidrug resistance protein (*MDR1)* was reported [7]. A CTR1-mediated uptake of intestinal Cu was shown in mice [8]. Cu inside the cell is distributed to other cell compartments, like mitochondria or via *ATOX1* to the trans-Golgi-network (TGN). At the TGN, *ATP7B* provides Cu for incorporation into enzymes, e.g. CP and hephaestin (*HEPH*) in the intestine. Metallothionein (MT) is a low molecular metal-binding protein that is essential for the intracellular homeostasis of various metals resulting in cytoprotection and detoxification [9]. Excess Cu affects the intracellular localization of ATP7B and leads to vesicular storage or ultimately to excretion via bile [10]. ATP7A delivers Cu to lysyl oxidase, tyrosinase and peptidyl alpha-monooxygenase. In the enterocyte, ATP7A is commonly believed to represent the main Cu exporter, leading to the release of Cu into the blood for whole body distribution. Absence of *ATP7A* was shown to increase the intracellular accumulation of Cu in intestinal cells [11]. *ATP7B* is also expressed in enterocytes [12], however it´s functional role in human intestinal cells is largely unexplored and most evidence was previously derived from WD animal models. Lower Cu concentrations were observed in duodenal tissue of *Atp7b*^*-/-*^ mice as compared to wildtype suggesting that functional loss of *ATP7B* results in decreased uptake/storage [13, 14]. Pierson *et al*. suggested that Atp7b mostly resides in the vesicular compartment suggesting a role in the cytosolic storage of Cu in intestinal cells rather than in the export of Cu as observed in the liver. Furthermore, a crosslink of iron and Cu homeostasis was previously suggested [15].

Cu loading of cells was implicated to affect lipid metabolism. Low hepatic Cu levels were correlated with liver steatosis in nonalcoholic fatty liver disease (NAFLD) [16]. In contrast, WD patients revealed normal serum triglyceride (TG) and cholesterol levels, but a reduction of cholesterol after hepatic manifestation [17]. Of note, studies of WD animal models revealed changes of TG and cholesterol levels in liver and intestine [14, 18–20]. For enterocytes of *Atp7b*^*-/-*^ mice, an impact of ATP7B on the chylomicron production was recently suggested [14]. High dietary fat increases the chylomicron production of enterocytes, which transport TGs into lymph and blood [21]. The synthesis of lipoproteins in the intestine, e.g. chylomicrons, VLDL, and HDL, depends on the availability of specific lipids, structural apolipoproteins (e.g. ApoB48 and ApoE), and export supporting proteins, like ABCA1. Cu was proposed to interfere with several processes of lipid metabolism; however the determination of the Cu impact needs further work.

The purpose of our study was the generation of a human intestinal *ATP7B* KO cell line to study the interrelation of Cu and lipid metabolism at the level of the enterocyte.

## Materials and methods

### Cell culture

The human epithelial colorectal adenocarcinoma cell line Caco-2 was received from American Type Culture Collection (ATCC, Manassas, VA, USA). Caco-2 cell lines were grown in DMEM High Glucose (GE Healthcare, Chicago, IL, USA) supplemented with 10% fetal bovine serum (Gibco, Carlsbad, CA, USA) and 100 U/mL penicillin/streptomycin (Hyclone, Logan, UT, USA). For differentiation, 10^5^ cells were seeded on 24 mm diameter wells and grown to confluence for 14 days to allow cell differentiation [22]. Media change was performed 2–3 times a week. Cells were maintained in 5% CO_2_ at 37°C in a humidified chamber.

### CRISPR/Cas9 knockout

10^6^ Caco-2 cells were seeded in standard cell culture medium. The next day, cells were transfected with 2 μg of plasmid pSpCas9(BB)−2A-Puro (PX459) V2.0 (Addgene plasmid no. 62988 was a gift from Feng Zhang [23]) containing Cas9 endonuclease and gRNA scaffold with an *ATP7B*-specific sgRNA sequence (5’-ATATCGGTGTCTTTGGCCGA-3’). After 24 h of transfection, a single cell dilution was performed. Standard medium containing 1.5 μg/ml puromycin was added for 72 h for selection.

### Sequence analysis

DNA was purified using QIAamp DNA mini kit (Qiagen, Hilden, Germany). DNA sequencing was performed using Big Dye Version 3.1 (Life Technologies). For gross sequence analysis of the Caco-2 cell clones the primers 5’-AGAGGGCTATCGAGGCAC-3’ / 5’-GGGCTCACCTATACCACCATC-3’ were used. The respective PCR product derived from one clone (clone #1) was ligated into plasmid pCR2.1-TOPO (TOPO TA Cloning kit; Invitrogen). After transformation of the ligation mixture into *E*.*coli*, DNA from bacterial clones was isolated and forwarded to sequence analysis. The complete *ATP7B* coding region gene was also analyzed for Caco-2 cell clone #1 [24].

### siRNA knockdown

50 nM small interfering RNA (siRNA) directed against *ATP7A* (AM16708; Ambion, Foster City, CA, USA) and 4 μl RNAiMax (Gibco) were incubated with cells for 24 h. A scrambled oligonucleotide with an unrelated sequence was used for control.

### Retroviral transduction

Retroviral transduction was performed with vector pGCsamEN.ATP7B, expressing the coding region of human *ATP7B* using established protocols [24]. Selection of cells was performed with 1 μg/ml of blasticidin (Invitrogen) starting at day 1 after transduction.

### Viability assay

Cells were cultivated in phenol red free cell culture medium (Lonza, Basel, Switzerland). CuCl_2_ (Sigma-Aldrich, St. Louis, MO, USA) was added for 48 h. MTT (1mg/ml 3-[4, 5-dimethylthiazolyl-2]-2, 5-diphenyltetrazolium bromide; Sigma-Aldrich) incubation was for 2 h. Cells were solubilized with sodium dodecyl sulfate (SDS; Roth, Karlsruhe, Germany) and dimethyl sulfoxide (DMSO; Roth). Absorbance was measured at 560 nm and viability was calculated as percentage of untreated control cells (100%).

### Copper accumulation

0.1 mM CuCl_2_ was added for 24 h in standard cell culture medium. Cells were washed five times with PBS and the cell pellet was collected. For measurement of subcellular Cu fractions differential centrifugation was used as described before [24]. Cu concentrations were determined by atomic absorption spectroscopy (Shimadzu AA-6300, Kyoto, Japan). Bradford protein assay (BioRad, Hercules, CA, USA) was used to normalize Cu measurements.

### Triglyceride determination

0.1 mM CuCl_2_ and oleic acid (125 μM; O3008, Sigma-Aldrich) were added in standard cell culture medium for 24 h. Cells were subjected to TG quantification (Triglyceride Quantification Assay, ab65336, Abcam) or AdipoRed Assay Reagent (PT-7009, Lonza) according to the protocol of the manufacturer. Total protein concentration was used for normalization. Cell culture supernatant was collected and triglycerides were determined by an automated cobas 8000 analyzer system (Roche Diagnostics GmbH, Mannheim, Germany).

### Electron microscopy

Cells grown in standard cell culture medium were treated with 0.1 mM CuCl_2_ for 24 h. Cell pellets were resuspended in 2.5% glutaraldehyd in Sorensen’s Phosphate Buffer (0.133 M Na_2_HPO_4_, 0.133 M KH_2_PO_4_, pH 7.2) and fixed with 1% osmiumtetroxide. After dehydration, specimens were embedded and sixty nanometer ultrathin sections were prepared (Leica Ultra Cut E ultramicrotome, Vienna, Austria). Counterstaining was performed with uranyl acetate and lead. Samples were inspected on a transmission electron microscope EM 208S transmission electron microscope (Phillips, Hamburg, Germany). Measurement of lipid droplets sizes was performed using the software ImageJ according to the reference scale of each image. At least 20 cells were analyzed per condition from different experiments.

### Real-time quantitative PCR

Total RNA was isolated by RNeasy kit (Qiagen, Hilden, Germany). Transcription was performed using 1 μg of RNA and SuperScript III (Invitrogen, Carlsbad, CA, USA). For quantitative real time PCR (qPCR) SYBR Green PCR Core Plus (Eurogentec, Liège, Belgium) and primers (S1 Table) were added. PCR was analyzed on the ABI Prism 7900 HT Sequence Detection System (Applied Biosystems, Carlsbad, CA, USA). Ct values were normalized to the expression of the house-keeping gene *Actin* (ΔΔCt method) and log_2_ expression was calculated.

### Western blot

Cells were lyzed in RIPA buffer (60 mM Tris-HCl, 150 mM NaCl, 2% Na-deoxycholate, 2% Triton X-10, 0.2% SDS, and 15 mM EDTA) and protease inhibitors (Roche, Basel, Switzerland; Complete Mini, EDTA-free). 10 μg protein lysate was loaded on a 10% sodium dodecyl sulfate polyacrylamide gel (SDS-PAGE). Polyclonal anti-rabbit ATP7B antibody (1:1,000) was added overnight for detection (kind gift of I. Sandoval, Madrid, Spain). For protein loading control β-Actin was assessed (1:1,000; sc-47778 HRP, Santa Cruz Biotechnology, Santa Cruz, CA, USA).

### ApoE ELISA

ApoE protein was determined using Human ApoE ELISA Kit (EHAPOE, Thermo Scientific). Cell culture supernatants were used at 1:10 dilution and absorbance was assessed at 450 nm. Normalization was performed by total protein concentration.

### Statistical analysis

Statistical analysis was performed by Kruskal-Wallis 1-way ANOVA and Wilcoxon Mann-Whitney-test using SPSS 22.0 software and GraphPad Prism 8. A p<0.05 value was used to indicate significance.

## Results

### Generation of a human *ATP7B* CRISPR/Cas9 knockout intestinal cell line

**Fig 1:**
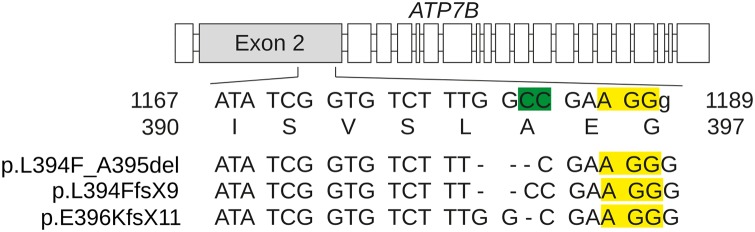
CRISPR/Cas9 target sequence for knockout of *ATP7B*. The nucleotide sequence of the CRISPR sgRNA that targets exon 2 of *ATP7B* is given in capital letters. The putative Cas9 cleavage site (green) and the PAM motif (yellow) are depicted. The amino acid sequence is also represented. Numbers refer to nucleotide and amino acid positions of *ATP7B*. The three compound deletions observed in the KO cell line are depicted.

An *ATP7B* knockout cell line was generated in human intestinal Caco-2 cells to study the impact of *ATP7B* on Cu and lipid metabolism. Caco-2 cells were transfected with a plasmid containing the endonuclease Cas9 and a guide RNA, targeting exon 2 of human *ATP7B* (Fig 1). Cell growth was observed in 13 clones after puromycin selection of CRISPR/Cas9 plasmid. Clones were characterized by gross sequence analysis of *ATP7B* exon 2 and subjected to MTT assay (S2 Table). Seven clones showed wildtype *ATP7B* and an almost identical viability as compared to parental cells when exposed to a toxic concentration of Cu (0.25 mM). Six clones showed significant reduced viability (<30%) and an ambiguous nucleotide sequence close to the PAM region after gross sequence analysis suggesting multiple compound deletions (S1A and S1B Fig, respectively). The cDNA of one Caco-2 cell clone (clone #1) was therefore further analyzed via bacterial cloning of the *ATP7B* exon 2 PCR product. Sequence analysis of the bacterial clones (n = 19) suggested a compound mutation consisting of altogether three deletions (p.L394F_A395del, p.L394FfsX9 and p.E396KfsX11) corroborating the reported polyploidy of the Caco-2 cell line (S3 Table) [25]. Importantly, a wildtype sequence could not be observed in the bacterial clones. The respective Caco-2 cell line was termed *ATP7B* KO (KO cells). The remaining coding sequence of *ATP7B* was unchanged suggesting that CRISPR/Cas9 mutagenesis induced a highly specific knockdown.

### *ATP7B* knockout cells display disturbed copper homeostasis

**Fig 2:**
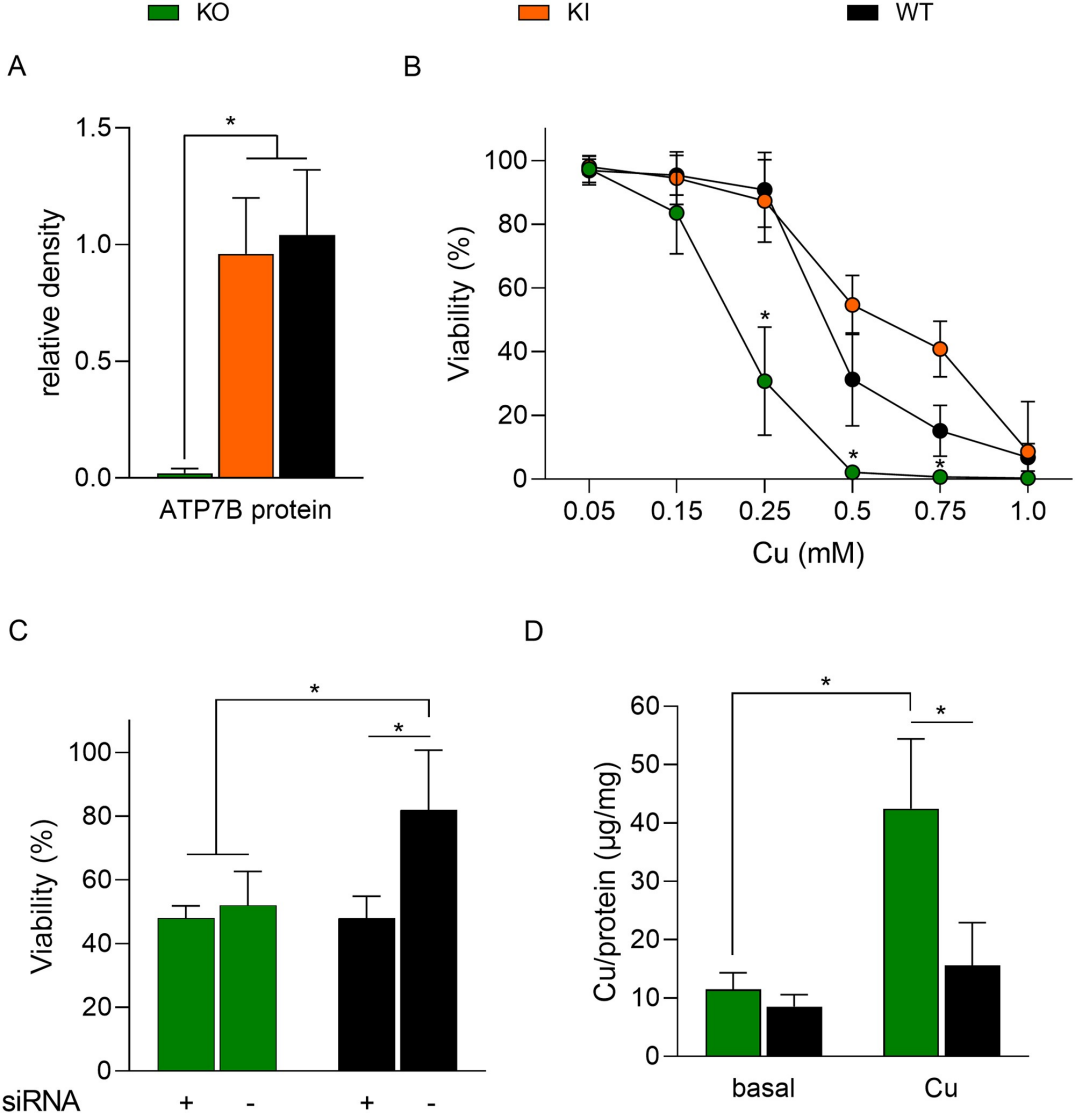
Knockout of *ATP7B* reduces intestinal Cu export. (A) Densiometric quantification of ATP7B protein expression following Western-blot analysis. Parental cells (WT) and *ATP7B* knockin cells (KI) were used as control. Mean ± SD are given (n = 3). (B) Cell viability was measured by MTT assay after 48 h of Cu exposure. Untreated cells were used as control (100%). Mean ± SD are given (n = 3–6). (C) Cells were treated with siRNA directed to *ATP7A* (+). Viability was determined after incubation with 0.25 mM Cu for 48 h. Scrambled siRNA treated cells (-) served as control. Untreated cells were set to 100%. Mean ± SD are given (n = 4–6). (D) Determination of intracellular Cu. Cells were exposed to standard cell culture medium (basal) or to 0.1 mM Cu for 24 h. Mean ± SD are given (n = 4). * P<0.05.

The expression of ATP7B protein was determined in KO cells to evaluate the effect of deletions in exon 2. A knockin cell line, termed KI cells, expressing the wildtype *ATP7B* ORF was established from KO cells by retroviral vector. An ATP7B-specific protein band could not be detected by Western blot analysis of KO cells (S2 Fig). KI and parental cells showed similar high levels of ATP7B expression as determined by densitometric quantification of ATP7B-specific protein bands (Fig 2A). In order to characterize the sensitivity of KO cells to high Cu, different Cu concentrations were exposed to the cells. A significant drop of viable cells was observed at Cu concentrations ranging from 0.25 mM to 1.0 mM in KO cells as compared to parental and KI cells (Fig 2B). Exposure to Cu concentrations above 0.5 mM resulted in an almost complete functional loss to evade Cu toxicity. In contrast, *ATP7B* knockin could fully restore survival suggesting that the CRISPR/Cas9 induced deletions are causative of the functional loss observed in KO cells. Since Caco-2 cells also express *ATP7A*, which was proposed to be a Cu exporter [26, 27], we also assessed whether knockdown of *ATP7A* in KO cells further impairs the function to evade toxic Cu. siRNA resulted in efficient knockdown of *ATP7A* mRNA (S3 Fig). *ATP7A* knockdown did not modulate viability of KO cells (Fig 2C). However, the viability of parental cells was significantly affected by *ATP7A* knockdown to the same level of KO cells (48%±3 and 48%±2, respectively).

The intracellular Cu storage of KO cells was determined (Fig 2D). When cells were propagated in standard cell culture medium (basal medium contained <2.5 μM Cu), an almost identical level of intracellular Cu was observed in KO and parental cells. However, KO cells revealed a significant increased level of intracellular Cu when incubated with 0.1 mM Cu for 24 h. Note, that cells cultivated at this Cu concentration showed no signs of apoptosis and proliferation arrest. After ultracentrifugation, most Cu (~80%) was observed in the supernatant of 15,000 xg fraction of the cell lysates suggesting low molecular weight, cytosolic storage (S4 Fig).

As Cu and iron homeostasis seems to be related [15], the sensitivity to toxic iron was determined in KO cells. In contrast to the reduced Cu sensitivity of KO cells, exposure to iron (10 mM to 30 mM) did not alter the cellular viability as compared to parental cells (S5 Fig).

### Modulation of gene expression after intestinal *ATP7B* knockout

**Fig 3:**
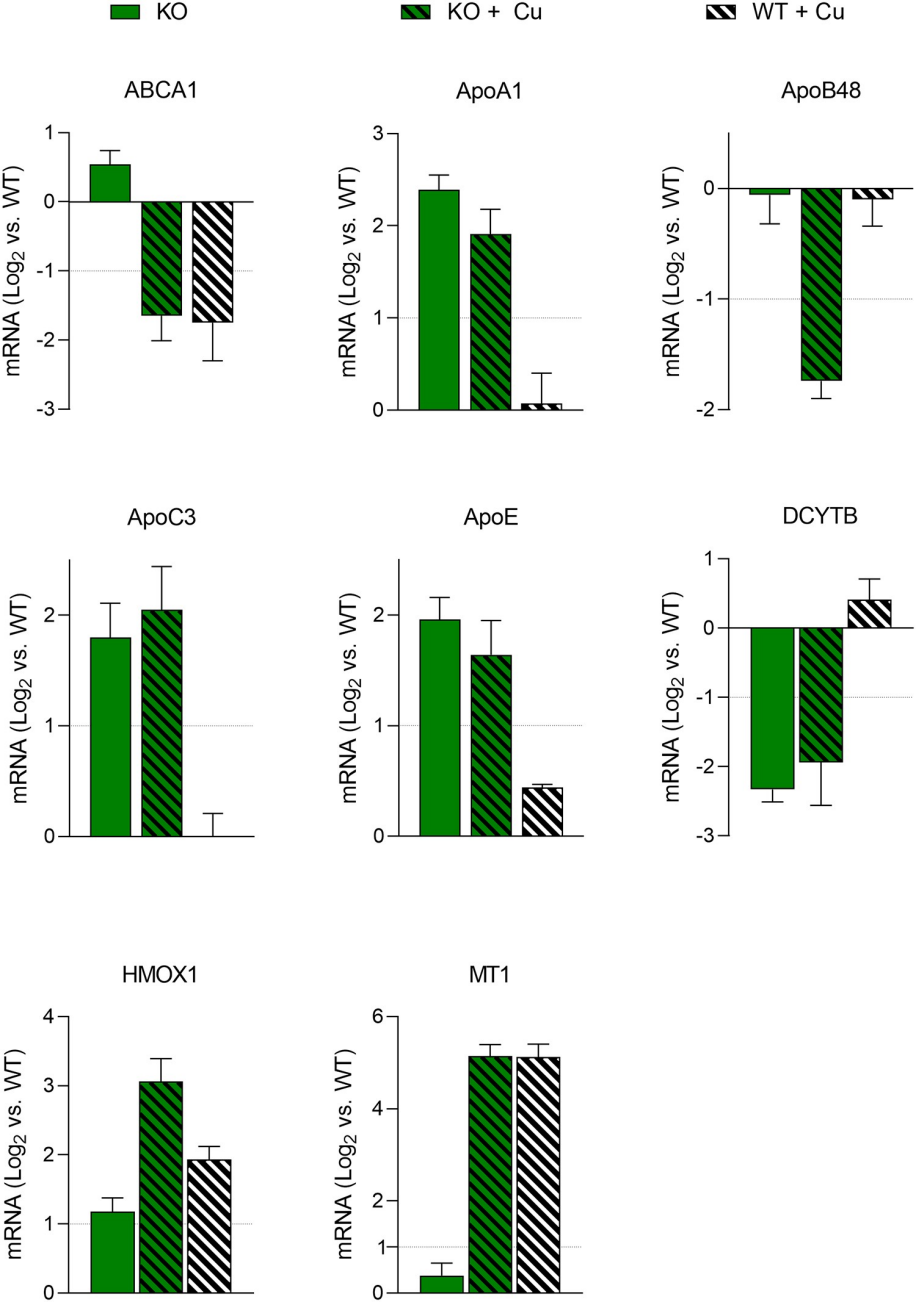
Modulation of gene expression following *ATP7B* knockout of intestinal cells. Real-time RT-qPCR analysis of cells grown in standard cell culture medium (basal medium) or treated with Cu for 24 h (stippled). A threshold of log_2_ expression ± 1 as compared to untreated parental cells was considered to indicate significance (dotted line). Mean ± SD are given (n = 3–6).

The question was addressed whether expression of genes related to Cu and lipid homeostasis was affected in the KO cell line. A set of 27 genes were selected from PubMed. Gene expression was analyzed by RT-qPCR analysis. Cells were grown in standard cell culture medium (basal medium) or incubated with 0.1 mM Cu. Expression was compared to parental Caco-2 cells grown in basal medium (control). 19/27 genes did not show significant changes of gene expression (≥ 1-log_2_ expression relative to control) regardless whether treated with Cu or not (S4 Table). Genes not modulated were related to Cu metabolism (*ATP7A*, *ATOX1*, *CTR1*, *MTF1* and *SOD1*), iron metabolism *(DMT1*, *EPAS1*, *FPN1*, *HEPH*, *MRP1* and *STEAP3)*, lipid metabolism (*HMG-CoA*, *LDLR*, *PLN2*, *PPARα*, *PPARγ*, and *VLDLR*), and apolipoprotein synthesis *(ApoA4* and *ApoB100*). In contrast, expression of three genes involved in protection from oxidative stress, metallothionein 1 (*MT1*), duodenal cytochrome B metal reductase *(DCYTB)*, and hemeoxygenase 1 *(HMOX1)*, were affected in KO cells (Fig 3). After addition of Cu, *MT1* was significantly induced, however to similar high levels as compared to parental cells. *DCYTB* was significantly downregulated in KO cells regardless of Cu addition. *HMOX1* expression was induced in KO cells with highest levels after addition of Cu, suggesting that a high anti-oxidative response is induced after knockdown of *ATP7B* in the intestinal cell line. Of note, five genes related to lipid and apolipoprotein synthesis were modulated in KO cells (Fig 3). *ABCA1*, a cholesterol efflux pump belonging to the ATP-binding cassette (ABC) transporters [28], was significantly downregulated in KO and parental cells after Cu treatment. *APOA1*, *APOC3* and *APOE* were upregulated in KO cells regardless whether or not Cu was exposed to the cells. *APOB48* was downregulated in KO cells after Cu treatment, whereas parental cells receiving the same treatment did not show modulation of mRNA level as compared to control.

### *ATP7B* knockout affects lipid metabolism in intestinal cells

**Fig 4:**
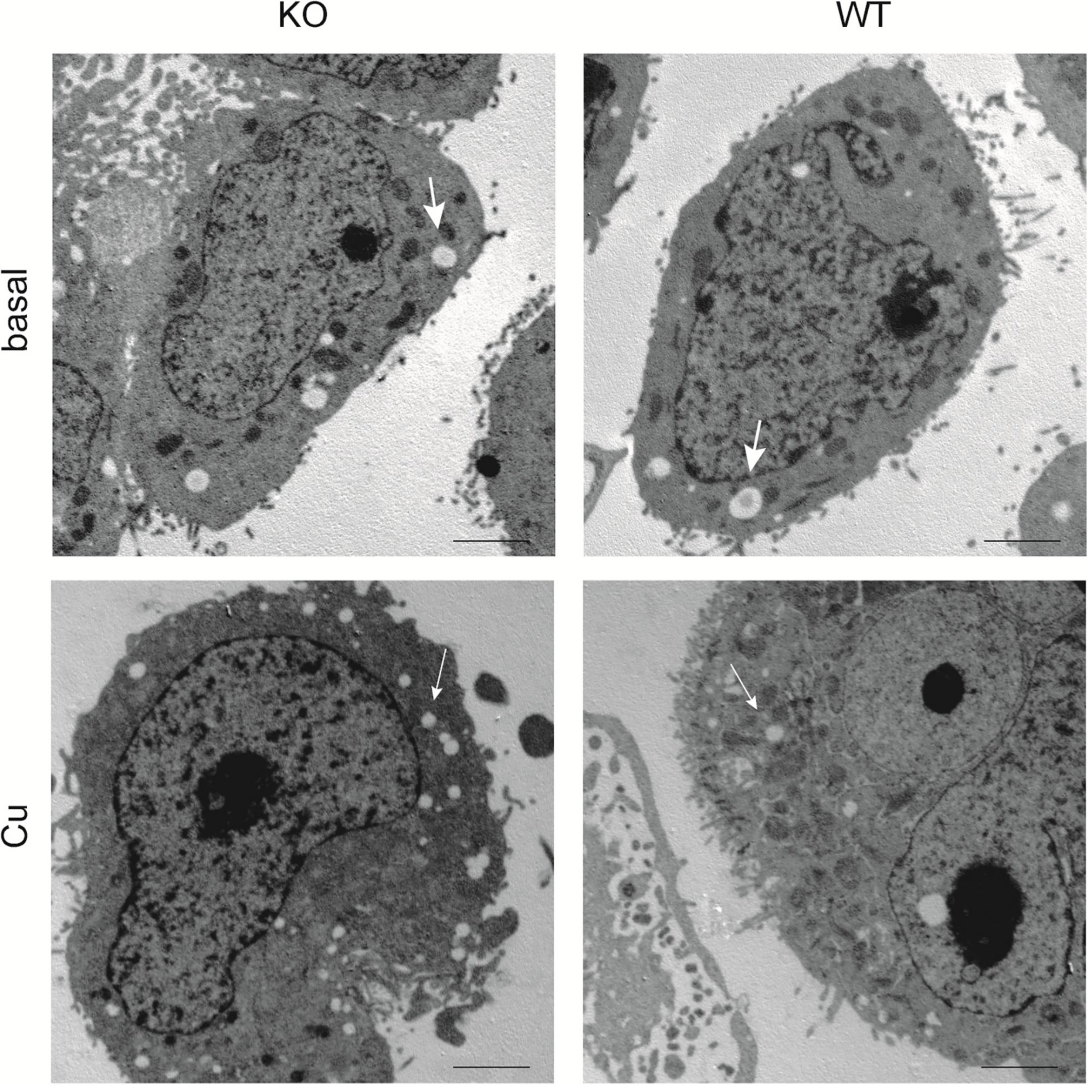
Accumulation of lipid droplets in KO cells. Electron microscopy images of KO and parental cells (WT) are shown. Large sized (bold arrows) and small sized LDs (plain arrows) are depicted. Cells were incubated in basal medium (top) and following Cu treatment (bottom). Note, accumulations of small sized LDs after Cu treatment. One representative experiment of six is shown. Scale bar, 2 μm.

Cu transport was proposed to affect lipid metabolism of intestinal cells. The number of intracellular lipid droplets (LDs) was investigated in KO cells by electron microscopy (Fig 4). According to their size, two classes of LDs were defined, having large (>600 nm) and small diameters (<600 nm). The numbers of LDs were similar in KO and parental cells when cultivated in standard cell culture media (basal media contains ~10 mg TG/dl) (Table 1). However, addition of Cu (0.1 mM) increased (~3-fold) the number of smaller sized LDs in KO cells and to a somewhat lesser extent (~2-fold) in parental cells. In parallel, the number of larger sized LDs decreased (~3-fold) in both cell lines after addition of Cu suggesting that maturation of large sized LDs is impaired by Cu.

**Table 1:** Number of lipid droplets in KO cells.

	WT	KO	WT + Cu	KO + Cu
Size (<600nm)	3.0 (292.0 ±35)	3.9^T^ (287.0±35)	6.2* (256.0±18)	13.2* (230.8±22)
Size (>600nm)	3.2 (786.6±71)	4.4^T^ (753.4±62)	1.1* (808.0±14)	1.2* (732.7±38)

Numbers of LDs per cell and mean ± SD of LD diameter (nm; in parenthesis) are given.

*significance (P<0.05) vs parental cells (WT cells)

^T^not significant (P>0.05) vs parental cells (WT cells)

**Fig 5:**
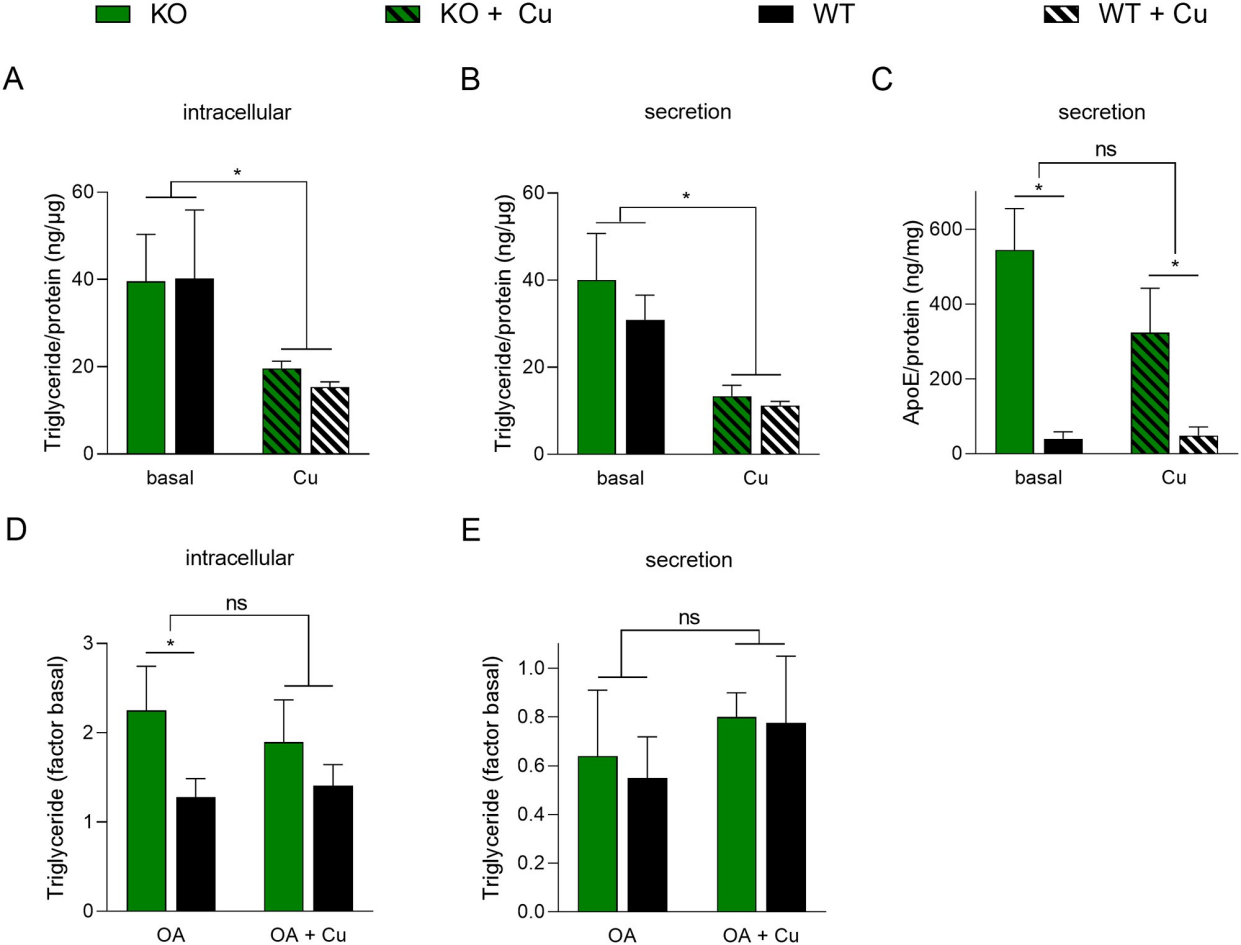
Impairment of triglyceride metabolism in intestinal cells after *ATP7B* knockout. (A) Intracellular TG levels of cells cultivated in basal cell culture medium and after Cu treatment. Mean ± SD are given (n = 3–5). (B) Determination of secreted TG levels in the supernatants of cell cultures cultivated in basal medium and after Cu treatment. Mean ± SD are given (n = 3–6). (C) Level of ApoE in the supernatants of cell cultures cultivated in basal medium and after Cu treatment. Mean ± SD are given (n = 3–4). (D) Intracellular TG levels after exposure of cells with oleic acid (OA). Levels are recorded relative to basal medium. Mean ± SD are given (n = 5). (E) Determination of secreted TG levels in the cell culture supernatants after OA treatment of cells. Mean ± SD are given (n = 5). * P<0.05.

In order to assess the storage and secretion of lipids in the cells, the concentrations of intracellular and secreted TGs were determined (Fig 5). Levels of intracellular and secreted TGs were similar in KO cells and parental cells when grown in basal medium (Fig 5A and 5B). Addition of Cu repressed intracellular TG storage and secretion. Secretion of ApoE, a determinant of various lipoproteins, was secreted at significant higher levels by KO cells as compared to parental cells (Fig 5C). Oleic acid (OA), a common long chain unsaturated fatty acid, was used to further investigate lipid metabolism of KO cells. After OA exposure, KO cells significantly accumulated higher amounts (~2-fold) of intracellular TGs as compared to parental cells (Fig 5D). However, total secretion of TGs was similar in KO cells and parental cells after OA addition, suggesting that high intracellular lipid storage does not implement an TG export of similar levels when *ATP7B* is lacking (Fig 5E). Addition of Cu did not affect lipid storage and secretion of parental cells following OA treatment.

## Discussion

Cu is an important molecule of many basic metabolic processes, including inflammatory response, antioxidant defense, and lipid peroxidation. The availability of Cu was recently linked to lipid metabolism and implicated in the pathogenesis of epidemic nutritional disorders, e.g. nonalcoholic fatty liver disease (NAFLD) and obesity [16, 29, 30]. In this scenario, the enterocyte of the proximal intestine has a central role to accommodate the body’s specific physiologic needs, including the uptake and processing of Cu and lipids. We explored the impact of copper transporter *ATP7B* to mediate Cu and lipid metabolism in the enterocyte, a role that was previously characterized for the hepatocyte [18, 19, 31]. Using CRISPR/Cas9 technology, a novel KO cell line was established from Caco-2 cells, one of the most studied human model with many biochemical and morphological characteristics of enterocytes [22, 32].

The CRISPR/Cas9 mutational approach targeting exon 2 of *ATP7B* resulted in the generation of several cell clones carrying compound heterozygous mutations around amino acid position 395, the targeted putative Cas9 cleavage site. In contrast to a previously reported mutational approach of *ATP7B*, a homozygous mutation was not observed here [33]. However, only a small number of cell clones was analyzed following an undirected mutational approach. While the deleterious variants observed in the KO cell line were not previously reported from patients, exon 2 is known to harbor numerous disease-causing mutations. Nevertheless, all variants of the KO cell line caused deletion/frame-shift mutations and resulted in absence of ATP7B protein expression and impaired ATP7B function.

Functional loss was indicated by Cu sensitivity of the KO cells, which could be specifically restored after re-expression of wildtype sequence. A significant elevation of intracellular Cu was observed in KO cells after exposition to 0.1 mM Cu suggesting that intracellular homeostasis is impaired in the situation of elevated Cu. The daily Cu intake by food can significantly vary and is estimated to be around 2 mg for a typical adult, suggesting that the Cu exposure of cells used in this study is within physiological ranges. Most Cu was found in the lower molecular weight fraction of the enterocyte cell lysates indicating that intracellular Cu storage is likely associated with the cytosol. Of note, at basal Cu concentrations we did not observe a higher Cu accumulation in KO cells as compared to parental cells. This finding parallels previous observations of high Cu accumulation in cell models and WD liver suggesting that the export of Cu is impaired after loss of *ATP7B* [34–36]. For hepatocytes, it is commonly viewed that the main function of ATP7B is the transport of excess Cu into the bile [1]. The role of *ATP7B* for Cu homeostasis in the enterocyte is however far less understood as compared to the liver. Our determination of Cu resistance in the KO cell line are suggestive of an ATP7B exporter function in intestinal cells, however the Cu efflux was not directly assessed. In addition, while reversal of high Cu sensitivity was observed after *ATP7B* knockin, other mechanisms, e.g. increased vesicular Cu sequestration, could be envisaged to cause evasion from toxicity. In LEC rats, where *ATP7B* is nonfunctional due to a deletion, high Cu levels were found in the intestine following a Cu diet corroborating our findings [37]. In contrast, AAS analysis and X-ray fluorescence microscopy of intestinal cells suggested reduced Cu levels in *ATP7B* [13, 14, 38]. Differences of the Cu content in diets and cell culture media, methodology of Cu determination, and differentiation status of the intestinal cells may account for some of the controversial findings. Further work is however required to address the exact role of *ATP7B* in the intestine, e.g. during polarized Cu trafficking.

*MT1* belongs to the first line defense antioxidant and *HMOX1* is an important defender of oxidative stress [39–41]. Downregulation of *DCYTB*, a metal reductase, is considered as prevention of oxidative stress [42]. Expression values of these genes indicate that KO cells do not show elevated oxidative stress at low (basal) Cu concentrations, but respond to Cu treatment by induction of an oxidative stress defense mechanism that is not mounted in parental cells. Various other genes, implicated to maintain intracellular Cu homeostasis, were not modulated by the low/high Cu exposure of KO cells suggesting that the regulation of basic Cu transporters, importantly *ATP7A*, *CTR1*, *DMT1* and *SOD1*, is preserved under the experimental conditions used in the study. In duodenal samples of WD patients *ATP7A* was increased and *CTR1* was reduced, whereas expression of *DMT1* and *ATP7B* was unaffected suggesting that some genes may be regulated by additional factors in patients [43]. *ATP7A* was suggested as a major Cu exporter of enterocytes [2]. High intestinal Cu concentrations were observed in patients having Menkes disease and in IEC-6 intestinal cells [11, 44]. In this regard it is of interest that our simultaneously impairment of *ATP7A* and *ATP7B* did not synergistically increase the Cu sensitivity of Caco-2 cells suggesting that both transporters are involved in the escape from toxic Cu. This result is surprising, since the double knockout cells, which have no option to export Cu either by ATP7A or ATP7B, show the same resistance as cells with only one downregulated transporter. We did not assess gene expression and Cu accumulation in double knockout cells and it could be argued that other genes might be modulated, e.g. CTR1 is downregulated/endocytosed resulting in an overall reduced Cu loading. However, CTR1 mRNA was not downregulated in the KO cell line. Further experiments, also addressing expression levels of proteins are needed. Taken together, our data suggest that similar to liver, where *ATP7B* presents exporting functions, this is also operative in the enterocyte, extending previous findings in knockout mice [14].

Cu metabolism was suggested to affect iron homeostasis [11, 42, 45]. A modulation of genes related to iron homeostasis was observed for intestinal cells [11, 42]. Our results on gene expression related to iron homeostasis as well as toxicity of KO cells after iron exposure did not support a role of *ATP7B* in iron homeostasis. However additional studies, including intestinal double knockouts, are needed to determine the role of the two Cu transporters for regulation of iron homeostasis in the enterocyte.

In a portion of WD patients, alterations in serum TG/cholesterol and appearance of liver steatosis suggested interrelation of Cu homeostasis and lipid metabolism [1, 17, 18, 31]. To gain more insights in the molecular mechanism, different aspects of lipid metabolism were investigated in KO cells. One important finding of our study was the increased level of intracellular TGs in KO cells after exposition to OA, one of the most commonly found long-chain monounsaturated omega-9 fatty acids in nature. TG storage is proposed as a protective mechanism against fatty acid-induced lipotoxicity [46]. OA was previously shown to be readily taken-up by Caco-2 cells which resulted in high-efficient secretion of TG-rich particles, prominently chylomicrons and VLDLs [47–50]. In hepatic cells, OA induces synthesis of hepassocin (HPS), a cytokine implicated to be reduced in NAFDL [51]. It is therefore tempting to speculate that such pathways are involved in the establishment of NAFDL in WD patients. The overall level of TG secretion by KO cells was unchanged as compared to parental cells suggesting that the release of excess intracellular TGs is hampered in the absence of *ATP7B*. In skeletal muscle cells, OA was found to accelerate lipid oxidation in a SIRT1-PGC1alpha-dependent mechanism [52]. Although we did not address lipid oxidation in our study, our finding of increased TG accumulation in KO cells suggests that *ATP7B* is involved in OA-induced lipid metabolism, at least in the enterocyte.

Our findings of increased levels of lipoprotein gene expression, namely *ApoA1*, *ApoC3*, and *ApoE*, are suggestive of an increased lipoprotein secretion in the absence of *ATP7B*. The level of *ApoB100* expression, one prominent protein of various lipoprotein classes [53], was however not affected in KO cells. Of note, ApoE, found in highest quantities in VLDL, was induced in KO cells. Several studies suggested a direct link between Cu deficiency and dyslipidemia with increased concentrations of ApoE [54, 55]. Similarly, overexpression of ApoE resulted in a combined hyperlipidemia phenotype [56]. However, ApoE expression of Caco-2 cells might be higher as compared to intestine and the relative role of intestine-secreted ApoE is not known [57, 58]. It is however conceivable that secretion of lipoproteins, which prominently carry TGs rather than cholesterol, like VLDL, is increased in enterocytes in the absence of *ATP7B*. This is in line with findings of reduced cholesterol levels in animal models of WD [18, 20]; however contradicting results were reported by others [19].

Cu displayed significant effects on the processing of lipids in KO and parental Caco-2 cells. Whereas the number of large size LDs was reduced, numbers of smaller sized LDs were increased suggesting that maturation of small sized LDs to large size LD, including chylomicrons [59], is hampered by Cu regardless whether or not *ATP7B* was expressed. The Cu-induced effect on LD generation may not be directly related to *ApoB48* mRNA expression, since parental Caco-2 cells expressed normal levels. Lipid transporter *ABCA1*, reported to contribute to the production of HDL particles via release of phospholipid and free, unesterified cholesterol from the plasma membrane [28], was repressed by Cu in intestinal cells regardless of *ATP7B* expression. Downregulation of *ABCA1* might therefore represent an intestinal response to Cu that may manifest in WD patients resulting in low cholesterol [17, 20, 60]. We also found a reduced intracellular TG storage and secretion that was independent of *ATP7B* expression when Cu treatment of the cells was performed in basal cell culture medium. Exposition of intestinal cells with Cu may thus prevent overall cellular lipid uptake resulting in decreased TG storage and secretion. Interference of Cu absorption and long-chain free fatty acid has been observed in rats. It was speculated that Cu may directly bind to free fatty acids and/or interact with the glycocalyx of the intestine [61, 62]. In a situation of Cu deficiency, rats showed increased intestinal levels of TGs suggesting that low intestinal Cu can stimulate lipogenesis [63, 64]. In contrast, Cu treatment resulted in decreased intestinal TG storage of enterocytes corroborating the findings of this study [51]. Conversely, when a high fat diet was used for animals lacking *ATP7B*, increased hepatic enzymes for cholesterol synthesis but normal values of serum TGs and cholesterol were found [60]. Taken together, our findings reveal a crucial role of Cu and *ATP7B* in the storage, processing, and secretion of lipids in a human enterocyte model.

## Supporting information

S1 Fig *ATP7B* CRISPR/Cas9 treatment of Caco-2 cells. (A) Cu resistance of cell clones (green) was examined by MTT assay. Clone #1 revealed compound deletion and clone #2 harboured wildtype *ATP7B*. WT cells (black) were used as control. Viability of cells was determined relative to untreated cells (100%). Mean ± SD are given (n = 3). *P < 0.05. ns, not significant. (B) Gross sequence analysis of clone #1 before bacterial cloning showed ambiguous nucleotide sequences between position 1181 and 1185. Note, that nucleotide sequence could be analyzed up to a certain position, whereas thereafter the sequence was unreadable (N) due to deletions. The PAM motif is marked in yellow. Forward (top) and reverse (bottom) sequence analysis is depicted.
(DOCX)

S2 Fig ATP7B is not detected in KO cell line. Western Blot analysis of ATP7B protein expression in KO, KI and WT cells. β-Actin was used as loading control. One representative blot of five is given.
(DOCX)

S3 Fig *ATP7A* is downregulated after siRNA treatment. mRNA expression in KO and WT cells after 24 h incubation with siRNA directed against *ATP7A*. A scrambled siRNA was used as control. Dotted line indicates threshold of log_2_ expression at -1. Mean ± SE are given (n = 3).
(DOCX)

S4 Fig Subcellular copper fractions of KO and parental Caco-2 cells. Cells were loaded with Cu and forwarded to analysis of subcellular Cu fractions by differential centrifugation. Cu was measured by AAS and normalized by protein. Mean ± SD are given (n = 3). ns, not significant.
(DOCX)

S5 Fig Iron resistance was not altered by *ATP7B* KO of Caco-2 cells. Cell viability after iron treatment for 48 h was measured in KO and WT cells using MTT assay. Untreated cells (100%) were used as control. Mean ± SD are given (n = 3).
(DOCX)

S1 Table Primers used for RT-qPCR analysis. (DOCX)

S2 Table Number of cell clones after CRISPR/Cas9 treatment. (DOCX)

S3 Table Sequence analysis of Caco-2 ATP7B KO cell line after bacterial cloning. (DOCX)

S4 Table Gene expression analysis of KO cells before and after copper load. Genes related to the Cu, iron (Fe) or lipid metabolism were examined. Cells were analyzed before and after Cu exposure. Log_2_ gene expression is given relative to parental (WT) cells prior Cu treatment. Mean ± SE is given (n = 3).
(DOCX)

## Abbreviations

Cu: copper
KI: *ATP7B* knockin
KO: *ATP7B* knockout
MD: Menkes disease
NAFLD: nonalcoholic fatty liver disease
TG: triglyceride
WD: Wilson disease
