# Supplementary material for: ATP7B knockout disturbs copper and lipid metabolism in Caco-2 cells
Source: PLoS One. 2020 Mar 10;15(3):e0230025. doi: 10.1371/journal.pone.0230025 (PMC7064347; doi:10.1371/journal.pone.0230025)
Supplement: S1 Fig — (A) Cu resistance of cell clones (green) was examined by MTT assay. Clone #1 revealed compound deletion and clone #2 harboured wildtype ATP7B. WT cells (black) were used as control. Viability of cells was determined relative to untreated cells (100%). Mean ± SD are given (n = 3). *P < 0.05. ns, not significant. (B) Gross sequence analysis of clone #1 before bacterial cloning showed ambiguous nucleotide sequences between position 1181 and 1185. Note, that nucleotide sequence could be analyzed up to a certain position, whereas thereafter the sequence was unreadable (N) due to deletions. The PAM motif is marked in yellow. Forward (top) and reverse (bottom) sequence analysis is depicted. (DOCX) [file pone.0230025.s001.docx]

##
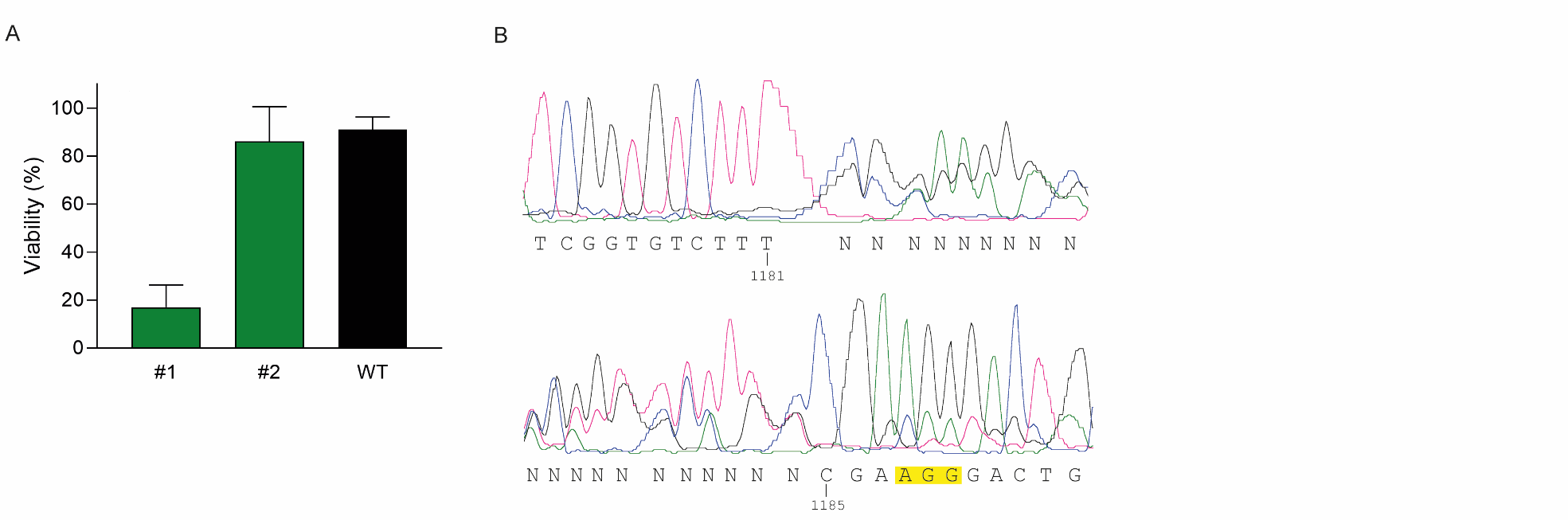


## S1 Fig. *ATP7B* CRISPR/Cas9 treatment of Caco-2 cells.

## (A) Cu resistance of cell clones (green) was examined by MTT assay. Clone #1 revealed compound deletion and clone #2 harboured wildtype *ATP7B*. WT cells (black) were used as control. Viability of cells was determined relative to untreated cells (100%). Mean ± SD are given (n=3). **P* < 0.05. ns, not significant.

(B) Gross sequence analysis of clone #1 before bacterial cloning showed ambiguous nucleotide sequences between position 1181 and 1185. Note, that nucleotide sequence could be analyzed up to a certain position, whereas thereafter the sequence was unreadable (N) due to deletions. The PAM motif is marked in yellow. Forward (top) and reverse (bottom) sequence analysis is depicted.
