# Supplementary material for: ATP7B knockout disturbs copper and lipid metabolism in Caco-2 cells
Source: PLoS One. 2020 Mar 10;15(3):e0230025. doi: 10.1371/journal.pone.0230025 (PMC7064347; doi:10.1371/journal.pone.0230025)
Supplement: S2 Fig — Western Blot analysis of ATP7B protein expression in KO, KI and WT cells. β-Actin was used as loading control. One representative blot of five is given. (DOCX) [file pone.0230025.s002.docx]

##
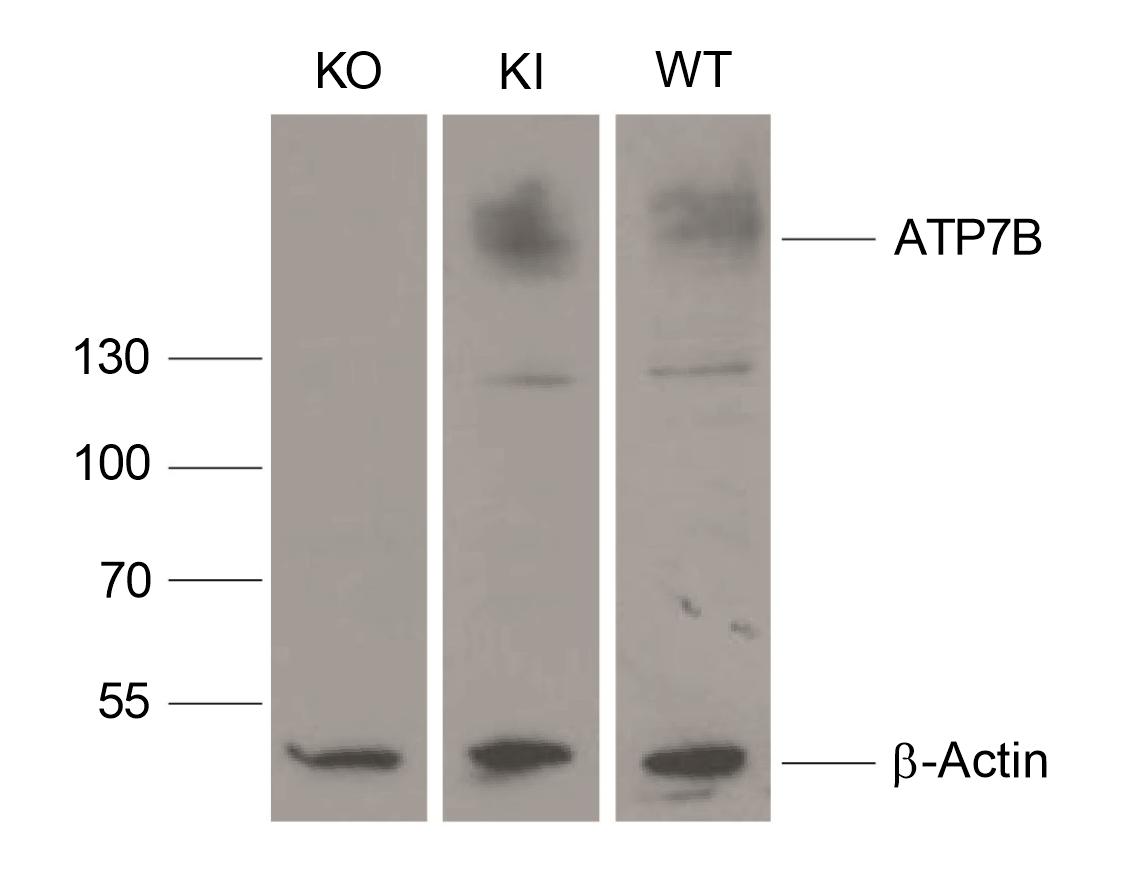


## S2 Fig. ATP7B is not detected in KO cell line.

Western Blot analysis of ATP7B protein expression in KO, KI and WT cells. β-Actin was used as loading control. One representative blot of five is given.
