# Supplementary material for: ATP7B knockout disturbs copper and lipid metabolism in Caco-2 cells
Source: PLoS One. 2020 Mar 10;15(3):e0230025. doi: 10.1371/journal.pone.0230025 (PMC7064347; doi:10.1371/journal.pone.0230025)
Supplement: S3 Fig — mRNA expression in KO and WT cells after 24 h incubation with siRNA directed against ATP7A. A scrambled siRNA was used as control. Dotted line indicates threshold of log2 expression at -1. Mean ± SE are given (n = 3). (DOCX) [file pone.0230025.s003.docx]

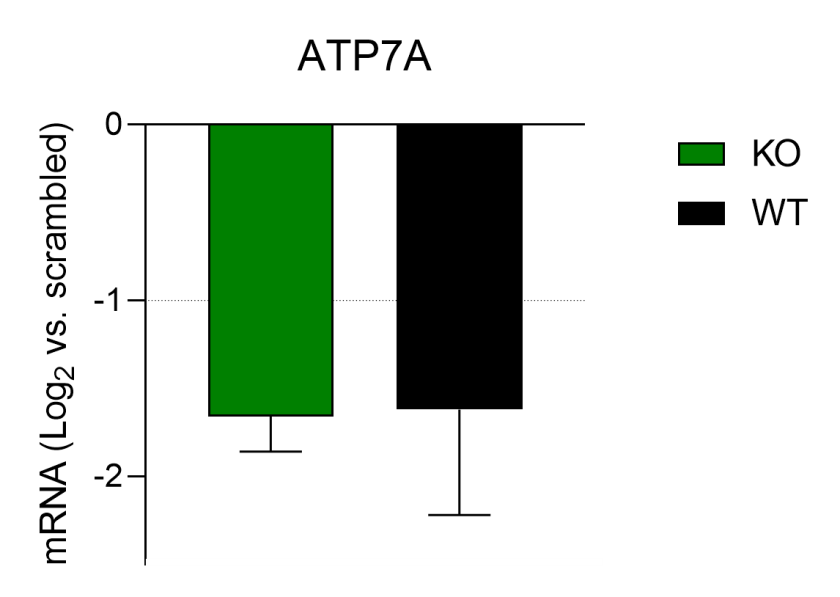


## S3 Fig. *ATP7A* is downregulated after siRNA treatment.

mRNA expression in KO and WT cells after 24 h incubation with siRNA directed against *ATP7A*. A scrambled siRNA was used as control. Dotted line indicates threshold of log_2_ expression at -1. Mean ± SE are given (n=3).
