# Supplementary material for: ATP7B knockout disturbs copper and lipid metabolism in Caco-2 cells
Source: PLoS One. 2020 Mar 10;15(3):e0230025. doi: 10.1371/journal.pone.0230025 (PMC7064347; doi:10.1371/journal.pone.0230025)
Supplement: S4 Fig — Cells were loaded with Cu and forwarded to analysis of subcellular Cu fractions by differential centrifugation. Cu was measured by AAS and normalized by protein. Mean ± SD are given (n = 3). ns, not significant. (DOCX) [file pone.0230025.s004.docx]

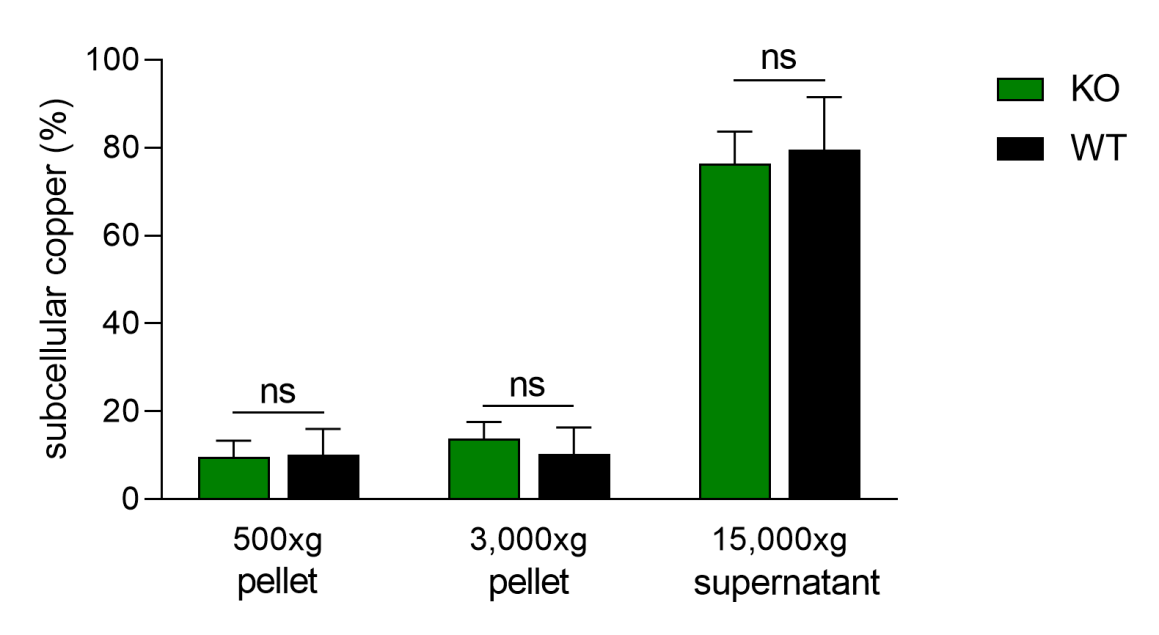


## S4 Fig. Subcellular copper fractions of KO and parental Caco-2 cells.

Cells were loaded with Cu and forwarded to analysis of subcellular Cu fractions by differential centrifugation. Cu was measured by AAS and normalized by protein. Mean ± SD are given (n=3). ns, not significant.
