# Supplementary material for: ATP7B knockout disturbs copper and lipid metabolism in Caco-2 cells
Source: PLoS One. 2020 Mar 10;15(3):e0230025. doi: 10.1371/journal.pone.0230025 (PMC7064347; doi:10.1371/journal.pone.0230025)
Supplement: S5 Fig — Cell viability after iron treatment for 48 h was measured in KO and WT cells using MTT assay. Untreated cells (100%) were used as control. Mean ± SD are given (n = 3). (DOCX) [file pone.0230025.s005.docx]

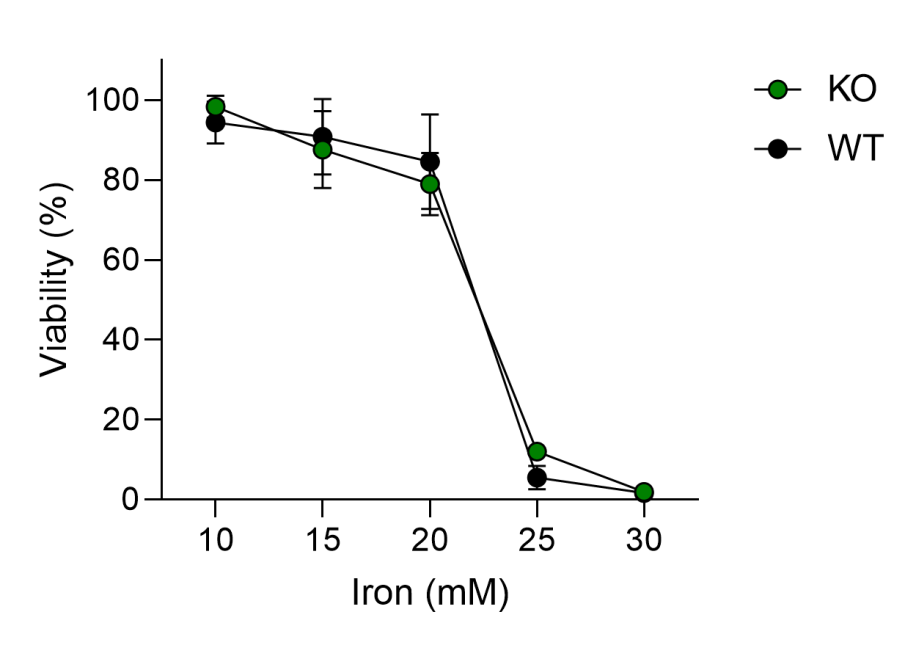


## S5 Fig. Iron resistance was not altered by *ATP7B* KO of Caco-2 cells.

Cell viability after iron treatment for 48 h was measured in KO and WT cells using MTT assay. Untreated cells (100%) were used as control. Mean ± SD are given (n=3).
