# Supplementary material for: ATP7B knockout disturbs copper and lipid metabolism in Caco-2 cells
Source: PLoS One. 2020 Mar 10;15(3):e0230025. doi: 10.1371/journal.pone.0230025 (PMC7064347; doi:10.1371/journal.pone.0230025)
Supplement: S1 Table — (DOCX) [file pone.0230025.s006.docx]

S1 Table. Primers used for RT-qPCR analysis.

| Gene Symbol | **Synonym** | **Accession Number** | **Forward/Reverse (5’-3’)** |
| --- | --- | --- | --- |
| ABCA1 | ATP binding cassette subfamily A member 1 | NM_005502 | GTTCAGGTGCCTTGGCAGTG/GGTTGGACCCTGCTATTCGTAC |
| Actin | Βeta-Actin | NM_001101 | CCGACAGGATGCAGAAGGAG/CACGGAGTACTTGCGCTCAG |
| ApoB100 | Apolipoprotein B | NM_000384 | AGGCATCTCCACCTCAGCAG/TGGAGAGGACTGAGGGCTGTAG |
| ApoB48 | Apolipoprotein B | NM_000384 | AGGCTGACTCTGTGGTTGACC/TGTGGCGTAGAGACCCATCAC |
| ApoA1 | Apolipoprotein A1 | NM_001318021 | GCTGGAGAGCTTCAAGGTCAG/ TATTCTGAGCACCGGGAAGG |
| ApoA4 | Apolipoprotein A4 | NM_000482 | GCAACTCAATGCCCTCTTCC/AAGGGCACCAGCTTCTTCTG |
| ApoC3 | Apolipoprotein C3 | NM_000040 | ACCAAGACCGCCAAGGATG/CAGGGAACTGAAGCCATCG |
| ApoE | Apolipoprotein E | NM_000041 | TGTCTGAGCAGGTGCAGGAG/GGCCTTCAACTCCTTCATGG |
| ATOX1 | ATX1 antioxidant protein 1 homolog | NM_004045 | CTGTGGAGGCTGTGCTGAAG/TCTTGTTGGGCAGGTCAATG |
| ATP7A | ATPase, Cu++ transporting, alpha polypeptide | NM_000052 | AGCAATGGCTGCTTCATCTG/GCAGGCAGTTCATAACTCTCG |
| CTR1 | Solute carrier family 31 member 1 | NM_001859 | TCGCTACAATTCCATGCCTGTC/TTGCAGGAGGTGAGGAAAGC |
| DCYTB | Cytochrome b reductase 1 | NM_024843 | TTCCCTGAGAGATCCTGCATAC/CCGAACACCAGGATCAGAAG |
| DMT1 | Divalent metal transporter 1 | NM_001174127 | GGGTTGGCAATGTTTGATTG/GCGTCCATGGTGTTCAGAAG |
| EPAS1 | Endothelial PAS domain protein 1 | NM_001430 | AGGTGTCAGGCATGGCAAG/GGCACGTTCACCTCACAGTC |
| FPN1 | Ferroportin 1 (SLC40A1) | NM_014585 | TGTCCCGGAGACAAGTCCTG/CAAAGGACCAAAGACCGATTC |
| HEPH | Hephaestin | NM_001130860 | TGCACTGCCATGTGACTGAC/TTGGTGATGACGGTGAGAGG |
| HMG-CoA | 3-hydroxy-3-methylglutaryl coenzyme A reductase | M11058 | TGCCATGGCTGGGAGCATAG/GTGCTGCATCCTGTCCACAG |
| HMOX1 | Heme oxygenase 1 | NM_002133 | GAGCTGCTGACCCATGACAC/GGGCAGAATCTTGCACTTTG |
| LDLR | Low density lipoprotein receptor | NM_000527 | GAGGGCTCTGTCCATTGTCC/AGCCGCCAGTTCTTCCATAG |
| MRP1 | ATP-binding cassette, sub-family C, member 1 | NM_004996 | ACTGCACCGTCCTCACCATC/GCCGTACTCCTGGATTTCTCC |
| MT1 | Metallothionein 1 | NM_005952 | CTCCTTGCCTCGAAATGGAC/GCATTTGCACTCTTTGCATTTG |
| MTF1 | Metal regulatory transcription factor 1 | NM_005955 | ACCAAGAACAAATTCAGCAAGC/ACACTGAGGCCAATCTGCTG |
| PLN2 | Perilipin 2 | NM_001122 | TGATGAGTCCCACTGTGCTG/GACTCAGCAGCTCCAGACCA |
| PPARα | Peroxisome proliferator activated receptor alpha | NM_001001928 | GGCTGCTATCATTTGCTGTGG/TCTGCAGGTGGAGTCTGAGC |
| PPARγ | Peroxisome proliferator activated receptor gamma | NM_001354667 | TGGAGCCCAAGTTTGAGTTTGC/CATTCAGCAAACCTGGGCGG |
| SOD1 | Superoxide dismutase 1 | NM_000454 | GTGGGCCAAAGGATGAAGAG/AATAGACACATCGGCCACACC |
| STEAT3 | STEAP3 metalloreductase | NM_001008410 | TCAGTCCTCACTGGGCTTTG/TGAAGGTGGGAGGCAGGTAG |
| VLDLR | Very low density lipoprotein receptor, transcript variant 1 | NM_003383 | CGCATGGGCCATTCTTCCTC/TGTGTTGCCAATTCCGCCAC |
