# Supplementary material for: ATP7B knockout disturbs copper and lipid metabolism in Caco-2 cells
Source: PLoS One. 2020 Mar 10;15(3):e0230025. doi: 10.1371/journal.pone.0230025 (PMC7064347; doi:10.1371/journal.pone.0230025)
Supplement: S2 Table — (DOCX) [file pone.0230025.s007.docx]

S2 Table. Number of cell clones after CRISPR/Cas9 treatment.

|  | No. wells |
| --- | --- |
| Seeding | 192 |
| Cell growth | 13 |
| WT | 7 |
| KO (compound) | 6 |
| KO (homozygote) | 0 |
