# Supplementary material for: ATP7B knockout disturbs copper and lipid metabolism in Caco-2 cells
Source: PLoS One. 2020 Mar 10;15(3):e0230025. doi: 10.1371/journal.pone.0230025 (PMC7064347; doi:10.1371/journal.pone.0230025)
Supplement: S3 Table — (DOCX) [file pone.0230025.s008.docx]

S3 Table. Sequence analysis of Caco-2 ATP7B KO cell line after bacterial cloning.

| **WT** | ATATCGGTGTCTTTGGCCGAAGG | mutation |
| --- | --- | --- |
| 1 | ATATCGGTGTCTTT---CGAAGG | p.L394F_A395del |
| 2 | ATATCGGTGTCTTT---CGAAGG |  |
| 3 | ATATCGGTGTCTTT---CGAAGG |  |
| 4 | ATATCGGTGTCTTT---CGAAGG |  |
| 5 | ATATCGGTGTCTTT---CGAAGG |  |
| 6 | ATATCGGTGTCTTT---CGAAGG |  |
| 7 | ATATCGGTGTCTTT---CGAAGG |  |
| 8 | ATATCGGTGTCTTT--CCGAAGG | p.L394FfsX9 |
| 9 | ATATCGGTGTCTTT--CCGAAGG |  |
| 10 | ATATCGGTGTCTTT--CCGAAGG |  |
| 11 | ATATCGGTGTCTTT--CCGAAGG |  |
| 12 | ATATCGGTGTCTTT--CCGAAGG |  |
| 13 | ATATCGGTGTCTTT--CCGAAGG |  |
| 14 | ATATCGGTGTCTTTGG-CGAAGG | p.E396KfsX11 |
| 15 | ATATCGGTGTCTTTGG-CGAAGG |  |
| 16 | ATATCGGTGTCTTTGG-CGAAGG |  |
| 17 | ATATCGGTGTCTTTGG-CGAAGG |  |
| 18 | ATATCGGTGTCTTTGG-CGAAGG |  |
| 19 | ATATCGGTGTCTTTGG-CGAAGG |  |

Exon 2 of 19 bacterial clones was sequenced after TOPO bacterial cloning of cDNA derived from KO cell line (clone #1). Each sequence represents the nucleotide sequence of one bacterial clone close to the PAM sequence (yellow) and putative Cas9 cleavage site (green). The amino acid mutation of each nucleotide sequence group is given.
