# Supplementary material for: ATP7B knockout disturbs copper and lipid metabolism in Caco-2 cells
Source: PLoS One. 2020 Mar 10;15(3):e0230025. doi: 10.1371/journal.pone.0230025 (PMC7064347; doi:10.1371/journal.pone.0230025)
Supplement: S4 Table — Genes related to the Cu, iron (Fe) or lipid metabolism were examined. Cells were analyzed before and after Cu exposure. Log2 gene expression is given relative to parental (WT) cells prior Cu treatment. Mean ± SE is given (n = 3). (DOCX) [file pone.0230025.s009.docx]

## S4 Table. Gene expression analysis of KO cells before and after copper load.

Genes related to the Cu, iron (Fe) or lipid metabolism were examined. Cells were analyzed before and after Cu exposure. Log_2_ gene expression is given relative to parental (WT) cells prior Cu treatment. Mean ± SE is given (n=3).

|  |  | KO |  | KO + Cu |  | WT + Cu |  |
| --- | --- | --- | --- | --- | --- | --- | --- |
|  |  | mean | SE | mean | SE | mean | SE |
| Cu metabolism | ATP7A | -0.93 | 0.19 | -0.70 | 0.42 | 0.47 | 0.12 |
|  | ATOX1 | -0.30 | 0.25 | 0.83 | 0.33 | -0.20 | 0.32 |
|  | CTR1 | -0.21 | 0.08 | -0.15 | 0.13 | -0.22 | 0.19 |
|  | MTF1 | -0.83 | 0.19 | 0.24 | 0.19 | -0.09 | 0.07 |
|  | SOD1 | -0.75 | 0.15 | -0.26 | 0.43 | -0.11 | 0.32 |
| Fe metabolism | DMT1 | -0.64 | 0.11 | -0.37 | 0.18 | -0.02 | 0.21 |
|  | EPAS1 | -0.84 | 0.14 | -0.37 | 0.12 | 0.06 | 0.23 |
|  | FPN1 | -0.15 | 0.19 | ND | ND | ND | ND |
|  | HEPH | -0.84 | 0.12 | -0.81 | 0.49 | -0.72 | 0.33 |
|  | MRP1 | ND | ND | -0.44 | 0.58 | 0.62 | 0.26 |
|  | STEAP3 | -0.31 | 0.24 | -0.65 | 0.55 | 0.73 | 0.40 |
| lipid metabolism | HMG-CoA | -0.05 | 0.20 | 0.01 | 0.31 | 0.01 | 0.19 |
|  | LDLR | 0.31 | 0.18 | 0.50 | 0.35 | 0.80 | 0.24 |
|  | PLN2 | -0.12 | 0.29 | -0.41 | 0.39 | 0.07 | 0.32 |
|  | PPARα | -0.27 | 0.32 | -0.55 | 0.22 | -0.07 | 0.21 |
|  | PPARγ | -0.62 | 0.36 | -0.18 | 0.32 | -0.19 | 0.25 |
|  | VLDLR | 0.32 | 0.22 | 0.87 | 0.26 | 0.03 | 0.20 |
| apolipo-  proteins | ApoA4 | 0.20 | 0.16 | 0.20 | 0.48 | -0.47 | 0.26 |
|  | ApoB100 | 0.45 | 0.32 | -0.40 | 0.43 | -0.24 | 0.43 |

ND, not determined
